# Scientific rewards for biomedical specialization are large and persistent

**DOI:** 10.1186/s12915-022-01400-5

**Published:** 2022-09-30

**Authors:** Gaétan de Rassenfosse, Kyle Higham, Orion Penner

**Affiliations:** 1 College of Management of Technology, Ecole polytechnique fédérale de Lausanne, Lausanne, Switzerland; 2 Institute of Innovation Research, Hitotsubashi University, Tokyo, Japan

**Keywords:** Scientific specialization, Scientific impact, Scientific careers, Science of science, Bibliometrics, Research systems

## Abstract

**Background:**

While specialization plays an essential role in how scientific research is pursued, we understand little about its effects on a researcher’s impact and career. In particular, the extent to which one specializes within their chosen fields likely has complex relationships with productivity, career stage, and eventual impact. Here, we develop a novel and fine-grained approach for measuring a researcher’s level of specialization at each point in their career and apply it to the publication data of almost 30,000 established biomedical researchers to measure the effect that specialization has on the impact of a researcher’s publications.

**Results:**

Using a within-researcher, panel-based econometric framework, we arrive at several important results. First, there are significant scientific rewards for specialization—25% more citations per standard deviation increase in specialization. Second, these benefits are much higher early in a researcher’s career—as large as 75% per standard deviation increase in specialization. Third, rewards are higher for researchers who publish few papers relative to their peers. Finally, we find that, all else equal, researchers who make large changes in their research direction see generally increased impact.

**Conclusions:**

The extent to which one specializes, particularly at the early stages of a biomedical research career, appears to play a significant role in determining the citation-based impact of their publications. When this measure of impact is, implicitly or explicitly, an input into decision-making processes within the scientific system (for example, for job opportunities, promotions, or invited talks), these findings lead to some important implications for the system-level organization of scientific research and the incentives that exist therein. We propose several mechanisms within modern scientific systems that likely lead to the scientific rewards we observe and discuss them within the broader context of reward structures in biomedicine and science more generally.

## Background

No researcher can be an expert in all fields. The entirety of human knowledge, even when considering a single field, is simply too much for a person to accumulate in a lifetime. Faced with this challenge, researchers specialize [1]. Through a series of decisions and choices, each researcher ends up accumulating the knowledge and skills necessary to advance some tiny sliver of the frontier of knowledge.

In addition to reducing the amount of knowledge and skills one must accumulate, specialization plays a key role in the sociology of science literature. In specializing, a researcher becomes a member of a community of researchers working on similar matters, in a similar fashion, with a similar viewpoint. It is within these specialized communities that the so-called invisible college is most visible [1, 2]. The concept of an invisible college has a long history—in this work, we work with the definition provided by Zuccala [3], which thinks of the invisible colleges as a group of actively interacting researchers that are drawn together to make progress within a specialized domain, often across institutional and geographical boundaries.

These observations present us with two points of view to consider the specialization of researchers. Viewed through the frontier-of-knowledge lens, specialization is required to reach the frontier of knowledge and start contributing within a human lifetime. Jones [4] formulates this as a “knowledge burden,” showing that as the frontier of knowledge expands more quickly, individuals reach it later in life. This observation holds for even the most lauded inventors and scientists in recent history [5]. Viewed through the lens of the invisible college, specialization determines the community of researchers within which the researcher falls—the group that one knows and is known to [2]. Research has shown that these weak ties play a crucial role in a variety of matters central to a researcher’s career [6, 7] and allow them to accumulate social capital [8].

### Measuring specialization and impact

While specialization is understood to play an essential role in research and researchers’ careers, most attention has been paid to how specialization emerges [9] and to the “content” of specialization [10], that is, the fields, sub-fields, and topics in which researchers choose to work. Here, we examine a very different aspect of specialization, namely the extent of specialization. In plain language, we capture how focused a researcher is on the topics they are working on the most. We then statistically examine the effect of specialization upon the researcher’s citation-based impact. Our operationalization allows a very fine-grained characterization of specialization, rather than one defined by journal classifications [11–13].

This operationalization of specialization is quite different from notions of monodisciplinarity. That is, the opposite of specialization, as defined here, is *not* interdisciplinarity—working at the intersection of fields is different from publishing work on many different topics—but rather generalization. A researcher working in a highly interdisciplinary space but publishing exclusively on the same small set of topics would be classified as a specialist within our framework. As such, our operationalization of specialization relates to the so-called “balance” dimension that is commonly referred to in the extant literature on interdisciplinarity [14, 15], though we note that this term is usually applied on the level of research outputs and not to researchers themselves [16, 17].

We use citations as a measure of impact for two primary reasons. First, while undoubtedly noisy and narrow in scope, citations remain a popular indicator of research impact [18], most perniciously in the context of career development. We note that the pervasiveness of citation-based averages is widely seen as a problem within scientometric circles [19, 20]. In this paper, we do not advocate for the use of citations as a performance metric generally, and we merely use them as an indicator of the level of attention an individual receives from their peers that may nonetheless form the basis for professional judgments during their research career [21]. Secondly, and relatedly, while it is unclear how well citation counts of individual articles correlate with conceptions of the intrinsic quality of those articles [22], we also expect that social status within a research community moderates the number of citations received [23, 24]. That is, in the framing of our research question and the interpretation of our results, we make the implicit assumption that scientific impact as reflected in citations are generated jointly by both judgment of research quality *and* citers’ perception of the author’s social status or ability, rather than solely as indicators of publication quality alone [25, 26], as we expect both of these factors to be moderated by one’s specialization level.

### The effect of specialization

Considering the frontier of knowledge point of view, it is unclear what effect the extent of specialization should have upon a researcher’s impact. From the “burden of knowledge” perspective [4], it is reasonable to expect that greater specialization will lead to a greater volume of output, particularly if one is working within teams with low coordination costs [27]. However, this reasoning does not necessarily extend to the impact of the output. A body of literature shows that combining bits of atypical [28] or less obvious [29] knowledge produces the highest impact papers. This literature would seem to indicate that as a researcher becomes more specialized and focuses more narrowly, they will produce lower impact research, as they will be sourcing ideas from an ever-smaller portion of the frontier of knowledge. However, collaboration can offset this effect, allowing the team as a whole to source ideas from any area in which a single member is specialized, no matter how narrowly the members specialize individually. As such, it is important to recognize that specialization on individual level, as a phenomenon, is distinct from disciplinarity on the level of particular outputs.

Viewed through the lens of the sociology of science and the invisible college, the expected professional benefits to specialization are more straightforward. Researchers can raise their visibility within a specific community more effectively by specializing to a greater extent, thereby attracting more citations. This line of thinking is consistent with one of the few previous studies using the extent of specialization that we are aware of [30]. In that work, the author finds that greater specialization leads to greater (financial) compensation for a sample of professors in linguistics and sociology. While the definition of specialization in that paper aims to capture the same concept as we do in the current work, the dependent variable does not capture the effect of specialization on individuals’ citation impact in a dynamic, within-researcher empirical setting, as used in the current work. Furthermore, our approach has been developed to take advantage of both the large numbers of keywords that can be applied to biomedical research, as well as the significant variation in the frequencies of these keywords assigned to different authors and publications. In a follow-on work [31], the authors incorporate a dynamic aspect to assess the various dynamic impacts of specialization on productivity and visibility within the fields of sociology and linguistics. Our work builds on this research, constructing a much finer-grain measure of specialization that is sensitive to topic popularity. Additionally, we apply the measure within the biomedical sciences—a very different research environment to that of sociology or linguistics. Indeed, our researcher fixed-effects econometric approach uncovers contrasting results to those of [31]; however, this may be due to the differences in publication and citation practices between biomedical fields and the social sciences.

A more recent work, using journal classifications to construct a researcher-level measure of interdisciplinarity, found that while more interdisciplinary researchers attracted more citations per paper, this effect was more than offset by lower productivity [11]. However, in that case, the researcher-level interdisciplinarity metric is explicitly designed to measure the diversity of fields from which a researcher draws upon for their research—it does not directly consider the prevalence distribution across these fields. In contrast, our definition measures the diversity of topics a researcher consistently works on. This difference in definition makes it difficult to compare our results directly, but research outside the sociology domain suggests that these research avenues may be complementary [16, 17, 32].

In this paper, we conduct an exploratory investigation into the scientific rewards associated with specialization using a dataset of 29,197 biomedical researchers. We estimate the extent to which a researcher is specialized using a novel measure that captures how intensely focused they are on specific topics. We measure scientific rewards in terms of *research impact*, proxied by citations per publication. In addition to measuring whether greater specialization positively or negatively affects a researcher’s impact, we also examine the role of career age, publishing rate, and recent changes in research topics in this relationship. Specifically, we answer four questions. First, does the extent to which a researcher is specialized affect the impact of their research? Second, does the effect of specialization on impact change with career age? Third, does the effect of specialization depend on the number of papers a researcher is producing? Finally, do recent changes in a individual’s research interests affect impact?

To provide answers to these questions, we need to use data that are rich in both longitudinal and cross-sectional dimensions, because we wish to measure the levels of specialization and impact at each point in a researcher’s career. To assess the trends that may be moderated by seniority or experience, we need to repeat these measurements over a long period. These considerations lead us to select a restricted cohort of nearly 30,000 well-published biomedical researchers. Each of these researchers is likely to be considered to be “successful” in the biomedical field by almost any measure. As such, all results are necessarily conditional on long-term success and should be interpreted with this caveat in mind. We discuss the details and implications of this restriction in the following sections. The “Methods” section describes the data, the variables, and the regression models.

## Results

Table 1 presents our main econometric results exploring the relationships between impact, specialization, career age, publishing rate, and changes in research orientation. We proxy the latter by a cosine similarity measurement that captures the extent of changes to a researcher’s topical interests since the previous observation period, as reflected in the subject matter of their published works. Column 1 shows the coefficients for a researcher fixed-effect panel regression model with no interaction terms. This baseline model indicates that a one standard deviation increase in specialization (calculated at the population level) results in a 25.7% increase in impact. That is, every paper that the researcher published in that time window receives on average 25% more citations than it would have otherwise (i.e., without the boost in specialization). Column 2 introduces the interaction term between specialization and career age, with the negative coefficient indicating that the rewards for specialization decrease over the course of a career. In other words, the payoff to specialization is lower for more experienced researchers, but it is never deleterious for the career stages we observe. Column 3 introduces the interaction term between specialization and yearly publication counts, with the negative coefficient indicating that the rewards for specialization decrease as a researcher publishes more.

**Table 1:** Fixed-effects panel regression results. Dependent variable is the log number of citations per paper, and the “specialization” variable is standardized. Standard errors are in parentheses. All control variables described in the text are included. Based on 29,197 unique biomedical researchers for a total of 213,019 researcher—time window observations

	(1)	(2)	(3)	(4)
Specialization	0.257***	0.361***	0.444***	0.732***
	(0.003)	(0.004)	(0.006)	(0.010)
Specialization × career age		− 0.007***		− 0.022***
Specialization × papers			− 0.061***	− 0.096***
Career age × papers				0.015***
Specialization × career age × papers				0.003***
Cosine similarity	− 0.404***	− 0.409***	− 0.437***	− 0.416***
	(0.013)	(0.012)	(0.012)	(0.012)
Control variables	Yes	Yes	Yes	Yes
Year fixed-effects	Yes	Yes	Yes	Yes
Researcher fixed-effects	Yes	Yes	Yes	Yes

***p < 0.0001

However, care should be taken in interpreting these coefficients: the benefits of specialization decrease as a function of career age and publishing rate, but the overall impact is always positive. That is, hypothetically increasing a researcher’s specialization appears to be associated systematically with a boost in impact. When the same hypothetical increase in specialization is applied to the same researcher later in their career (column 2) or for higher publishing rates (column 3), the boost in impact is less pronounced but always positive during the 35-year period we observe.

Lastly, column 4 shows the estimates for the complete interaction model. It includes the interaction between publishing rate and career age and the triple interaction term of specialization, publishing rate, and career age.

The variable cosine similarity maintains a negative impact on the average citation counts across all specifications. Thus, a researcher who makes greater changes in research direction from one period to the next achieves greater impact in the next period. This result, which is not causal, indicates that researchers are likely to reorient their research direction toward topics that result in higher future impact. Indeed, at least for our successful cohort, the ability of researchers to identify promising new avenues of research and immerse themselves in those areas may play a large role in the benefits of specialization we observe, adding color to previous work on the evolution of research interests [33]. More importantly in our context, this finding also provides some evidence against reverse causality of the relationship between specialization and impact—the idea that researchers may choose to specialize in topics that they expect to be more promising. The cosine similarity variable captures the changes in research topics and, therefore, picks up this effect. Thus, holding research topics constant, we do find strong positive returns to specialization.

As our observation periods are 3 years long, it is difficult to assess whether drastic changes in research direction on shorter timescales are rewarded with greater future impact, but recent work examining this question at the individual-paper level indicates that the reverse is likely true [34]. These contrasting results suggest that a balance between topic evolution and specialization is optimal for mid-to-long-term citation-based impact at the individual researcher level—at very short timescales, a sudden change in research interest appears to harm impact, while at longer timescales, a complete lack of topic evolution actively attenuates the benefits of specialization.

**Fig. 1:**
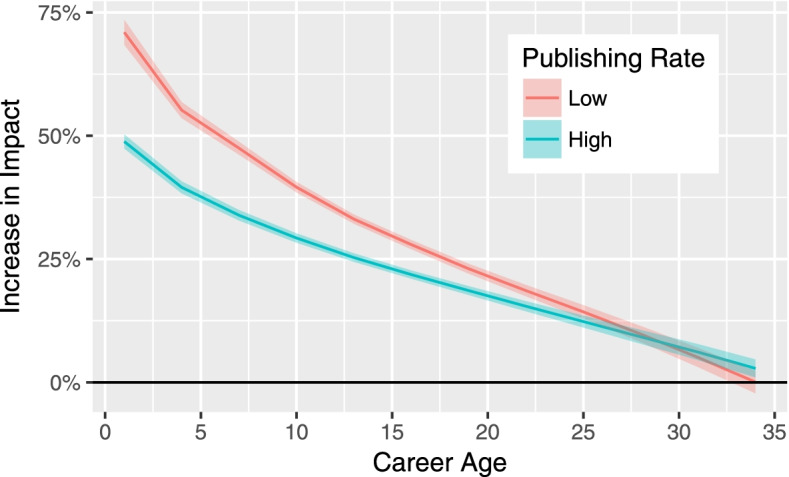
Increase in impact estimated for a one $\sigma$ increase in specialization as a function of career age, for two different publishing rates. Low publishing rate is estimated at the 12.5th percentile (middle of the first quartile) of papers per year. High publishing rate is estimated at the 87.5th percentile (middle of the fourth quartile) of papers per year. The shaded envelope of each line is the $99.9\%$ confidence interval. Based on 29,197 unique biomedical researchers for a total of 213,019 researcher-time window observations

Figure 1 provides a clear picture of the rewards for specialization—plotting the marginal effect (expected citation boost) for a one standard deviation increase in specialization as a function of career age for groups with a high or low publishing rate. These effects rely on the model found in column 4 of Table 1. The figure unambiguously answers the first three questions posed previously regarding the rewards for specialization. First, the returns to specialization are positive and significant, both in a statistical and a real-world sense. In the early stages of a career, these returns can exceed 70%, and even in mid-career, remain well above 10%. Indeed, in the early years of a researcher’s career, the returns to specialization can be great, boosting a typical researcher’s expected citations by 50% or more for a one standard deviation increase in specialization. Interestingly, the returns are never negative, regardless of career age or publishing rate. Second, the benefits associated with specialization decrease as a function of career age, regardless of publishing rate. Third, these benefits are higher for researchers with a lower publishing rate, although we observe a crossover around the end of the career in Fig. 1. However, the difference between the two groups after crossover is not statistically significantly different from zero.

### Robustness checks

To evaluate the validity of these results, we carry out a number of robustness checks, each described in more detail in the Appendix. First, we further analyze a sample of 22,577 biomedical researchers having published between 75 and 99 papers (rather than 100 or more) during their careers. We find quantitatively and qualitatively similar results. This finding gives us confidence that the results would hold for researchers having a lower publishing rate, although we cannot formally test this assertion because our measure of specialization would lose statistical power.

Second, we break the 100 or greater, and 75 to 99, publication samples into field-specific subsets and repeat the analysis on each of the eight most frequent fields found in our datasets. These fields include molecular and cell biology, medicine, neuroscience, gastroenterology, infectious diseases, radiology, nephrology, and psychology. In each of the eight fields, for each of the publication thresholds, we find that our main findings are qualitatively similar—however, the statistical significance is weaker for the smaller subfields.

Lastly, while our specialization measure is, by definition, conceptually distinct from interdisciplinarity, we also confirm that the measure is also empirically distinct. We split the cohort into monodisciplinary and interdisciplinary researchers (based on whether a researcher has published research in multiple sub-fields within the 3-year window). We then conduct several non-parametric statistical tests to detect any differences between the specialization distributions of these groups. We do not find any significant differences, providing evidence that our specialization measure is not sensitive to the differences in the levels of interdisciplinarity between researchers, at least at the sub-field level.

## Discussion

These results have significant implications for how we view and manage academic careers, especially in their fragile early stages. In the context of long-term success in biomedical research, working continuously on a relatively small and stable set of topics is rewarded by citations to the output of this research. Furthermore, there likely exists a complex web of interacting mechanisms that conspire to reinforce this phenomenon in ways that are not necessarily positive for the scientific enterprise. The remainder of this section will discuss these potential mechanisms, the limitations that may reduce the generalizability of this work, and open questions that may add more color to the current findings.

### The broader contexts of specialization

These findings also raise a great many questions. A critical one, of course, concerns the mechanisms that generate these rewards. From the frontier-of-knowledge point of view, it would seem that by focusing more intensely, a researcher may be in a better position to push the research frontier and attract more citations (perhaps by establishing priority on new findings). Taking the perspective of the sociology of science, one explanation is that by specializing more narrowly, a researcher may be better positioned to raise their visibility within a specific community. Indeed, the fact that we observe much greater rewards for specialization early in a researcher’s career (when a researcher has little reputation or visibility, to begin with) suggests that a young researcher’s impact is maximized by focusing on a community that is as specific as possible. However, the degree to which a researcher can “choose” a community at this early stage is unclear. We suggest that the choice of research direction likely comes before any intentional choice of community. The benefits diminish with career age could imply that it is easier to transfer visibility, accumulated advantage, or reputation across communities than it is to earn such things simultaneously across several communities.

It is also likely that current reward systems in contemporary science systems favor scientific specialization. While we draw a conceptual distinction between monodisciplinarity and specialization, there is almost certainly a strong relationship between the disciplinary nature of a particular scientific output and the level of specialization of the authors. The fact that science systems may punish interdisciplinary research [35, 36], due to perceived risk or otherwise, could nudge researchers to specialize or otherwise conform to more mainstream topics or career pathways [37]. Indeed, biomedicine often requires considerable resources to conduct high-impact, cutting-edge science. When the attraction of those resources for potentially risky projects is moderated by reputation, we expect to see the benefits of specialization decrease as a researcher becomes more established.

Our results are in line with prior work in the field of bibliometrics on so-called interdisciplinarity. That body of literature often breaks down interdisciplinarity into various components, most commonly variety, balance, and disparity [14]. While these concepts are usually applied to individual publications and have various operationalizations, we can make some useful comparisons between the current work and previous research in this area. In particular, the “balance” dimension generally attempts to capture the uniformity of the proportions of different component types that exist within some object of interest. For example, a bag containing candy in a variety of colors in roughly equal proportions would have a high balance, while one in which the vast majority are blue would have a low balance. This concept is often operationalized on the paper level using either the Shannon entropy or the reverse Gini coefficients of the set of disciplinary categories extracted from the references in each paper [16, 17, 32, 38, 39]. By categorizing interdisciplinarity into variety, balance, and disparity, it is possible to jointly estimate the effects of each of these concepts on impact. Within that framework, at the paper reference level, balance is almost universally *negatively* associated with impact in prior work, while the other two dimensions are either positively or ambiguously associated with impact. This nuanced point is very pertinent to the current work: the diversity of information sources and the evenness of the distribution of inputs across these sources have distinct and generally opposing relationships with impact. We note that the total number of unique Medical Subject Headings (MeSH) terms, a metric more related to the “variety” dimension, is included as a control in our main model. In sum, while it is not straightforward to directly compare the impact of individual articles with the impact of researchers more broadly, nor to compare operationalizations based on input knowledge with those based on research topics, our results are at least consistent with this prior research, although our researcher-focused approach allows for additional nuance arising from access to the temporal dimension.

Our analysis considers the quantity and broad patterns of collaboration by including control variables at the researcher level. However, the role of collaboration and division of labor in these results merits further discussion. An established body of literature shows that papers with a distinct interdisciplinary character [16, 17] or that arise from atypical combinations of knowledge [28] are more highly cited on average or more likely to represent a breakthrough than conventional disciplinary research. In the context of that literature, our results suggest that atypical combinations and interdisciplinarity may be best achieved by specialized researchers working together in teams. Although, there is likely a limit at which this hypothesis breaks down: as researchers become more specialized, they may have more difficulty communicating effectively, which in turn raises coordination costs [27, 40, 41].

### Limitations and open questions

Several outstanding issues moderate the practical usefulness of the results presented herein, especially in the context of academic careers. The first concerns the extent to which researchers can control their specialization actively. That is to say, how do the concrete day-to-day, project-to-project, and job-to-job decisions a researcher faces map on to specialization? Clearly, some subset of these decisions does affect specialization—for example, the selection of a specific new project. Researchers may perceive a risk of being “left behind” if they spend too much time working on projects outside of their usual specialized research path. These concerns raise an additional question, namely, if researchers do not control their own extent of specialization, who or what does? And in turn, how may those individuals, institutions, or systems craft policy to bring about a better configuration of the academic career in light of these findings? After all, we do not claim that the observed rewards associated with specialization are a good thing for science or society. The patterns we see are, in part, the result of a complex set of incentives and Matthew effects that are embedded in research ecosystems, many aspects of which have been heavily criticized for their various biases and inequitable outcomes [42–45]. While these incentives create an environment wherein specialization is a way for early-career researchers to “get ahead” in science, there is little to suggest that these incentives lead to efficient or equitable scientific progress. In fact, many prominent scholars suggest that the opposite is true for both individual researchers [35, 37, 46–48] and society-at-large [37, 46, 49, 50]. Furthermore, the relative inelasticity of research direction to funding [51] could entrench early-career specialization and exacerbate both opportunity costs and coordination costs associated with an over-specialized scientific workforce.

A second concern is the inherent selection bias in the above analysis that is necessary to obtain enough data for our method to be effective. The results in Table 1 pertain to researchers with at least 100 publications—our main sample comprises successful researchers by virtue of this threshold. While an alternative sample used as a robustness check includes those with between 75 and 99 publications, these researchers have achieved at least moderate success. The results for both samples are almost identical, which provides some evidence for the generality of these results, at least for biomedical researchers. However, even the 75-paper threshold may exclude many successful but less productive generalists [11]. At the same time, the interaction term between specialization and productivity in Table 1 indicates that higher productivity lowers total returns for a given level of specialization. As such, less productive specialists also reap greater reward for specialization. Therefore, our results are consistent with the idea that monodisciplinary and interdisciplinary specialists may *both* benefit from lower productivity at the individual output level. A more comprehensive investigation of the relationships between productivity, impact, and a researcher’s precise location on the specialization-disciplinarity plane may be a fruitful avenue for future research on this topic. While necessary for the operationalization of specialization used in this work, the imposed publication threshold implies that several lines of research are closed to us, such as the relationships between early specialization, academic career length, and opportunities outside of academia. For example, it may be the case that less-specialized high-impact early career researchers have a large selection of stable, well-compensated jobs outside of academia due to this breadth. In contrast, their specialized high-impact colleagues may have more choice of fellowships and positions within academia. In other words, funding systems and entrenched departmental structures at research institutions may act as filters in early academic careers, with a bias toward high-impact specialized researchers. Furthermore, it is possible that our results may be driven in part by Matthew effects [52]—a strong citation bias toward specialization could lead to specialized researchers remaining active and publishing for a longer period than less specialized researchers, leading to inclusion in our sample. In any case, it is clear that the benefits, or lack thereof, to producing a more or less specialized stream of research outputs remain a very pertinent aspect of career progression in science.

Finally, in the analysis presented above, we did not find a point after which increasing one’s extent of specialization becomes significantly deleterious in any discipline we examined (see Appendix for disciplinary breakdown). However, when taken to its absolute extreme, increasing specialization further may start to reduce a researcher’s impact. For example, by focusing exclusively on an extremely narrow subfield, a researcher may significantly limit the size of the audience for their work, effectively placing a low ceiling on the potential impact of any paper they publish. It is also entirely possible that the rewards for specialization break down in some periods, fields, or populations due to idiosyncracies in publishing, citation behavior, or institutional factors. This work focuses on biomedical researchers, and while one might expect similar results for other disciplines characterized by growing lab science and publishing rates (e.g., physics and chemistry), it is unclear whether specialization is universally beneficial across disciplines. For example, recent work suggests that the field-dependent speeds of the knowledge frontier could lead to disciplinary variation in the returns to specialization [53]—a generalist may find it more challenging to keep up with multiple fast-moving fields and identify new and salient knowledge recombination opportunities. However, it does stand to reason that the benefits of specialization may differ significantly for researchers in disciplines characterized by smaller teams and lower publishing rates, such as economics and mathematics. Further work is required to investigate how these dimensions of academic research affect the rewards for specialization.

## Conclusions

Given the magnitude of the scientific rewards to specialization presented in this article, it is critical to consider these benefits in the context of the rich and fast-moving discussion surrounding the academic career [37, 54]. The fact that the rewards for specialization are much greater early in a researcher’s career has clear implications for academic careers. Indeed, it is precisely in the early stages that such a career is the most fragile [55] while simultaneously being a period when researchers are expected to build a foundation from which they may explore and develop the ideas that may eventually yield research grants and permanent positions. Choosing to be more specialized early in one’s career not only boosts one’s impact during that period, but the early boost is compounded throughout the career by way of various Matthew effects [52, 56, 57]. The observation that rewards decrease with increased publishing also informs our understanding of the academic career. Researchers with limited resources are rewarded for maximizing their specialization and focusing on their core competencies.

Overall, this analysis points to significant scientific rewards to specialization in academic research in the biomedical context. Those rewards are significant in a statistical sense and magnitude—a 10 to 70% boost in the expected number of citations is entirely possible for an individual researcher given an increase in specialization of one standard deviation (depending on career stage). The rewards for specialization are most pronounced early in a researcher’s career and decrease monotonically thereafter. The benefits are greatest for researchers publishing at a lower rate relative to their cohort and decrease monotonically as the publishing rate increases. While the returns to specialization decrease with age and publishing rate, increasing one’s extent of specialization has no beneficial impact only at the longest times we observe, typically 25–35 years after a researcher first publishes. Lastly, changes in research direction at the scales of the time windows considered (3 years) appear to increase (within-researcher) citation-based impact.

## Methods

### Data

Our researcher dataset is drawn from the *Author-ity* disambiguation of PubMed [58, 59]. Each researcher in our sample meets three criteria. First, they published their first paper in 1975 or later. Second, they have published at least 100 publications up to the year 2009. Third, their primary area of research was determined to be biomedical using the algorithm outlined in the Appendix exploiting the journal clustering of Rosvall and Bergstrom [60]. To be explicit, we are not considering researchers publishing prior to 1975, publishing fewer than 100 papers (although this threshold is lowered to 75 papers in the Appendix), or primarily active in non-biomedical fields. For each researcher, we have a full publication record, which we cross reference from PubMed to the Clarivate Analytics Web of Science database. We analyze a total of 4,574,973 publications.

We broke each researcher’s career into 3-year windows and identified the papers they published during each period. From the papers published in each time window, we calculated our main explanatory variable, *specialization*, as well as additional explanatory and control variables, described below. This panel data was then analyzed using a researcher fixed-effect panel regression approach.

### Specialization measure

**Fig. 2:**
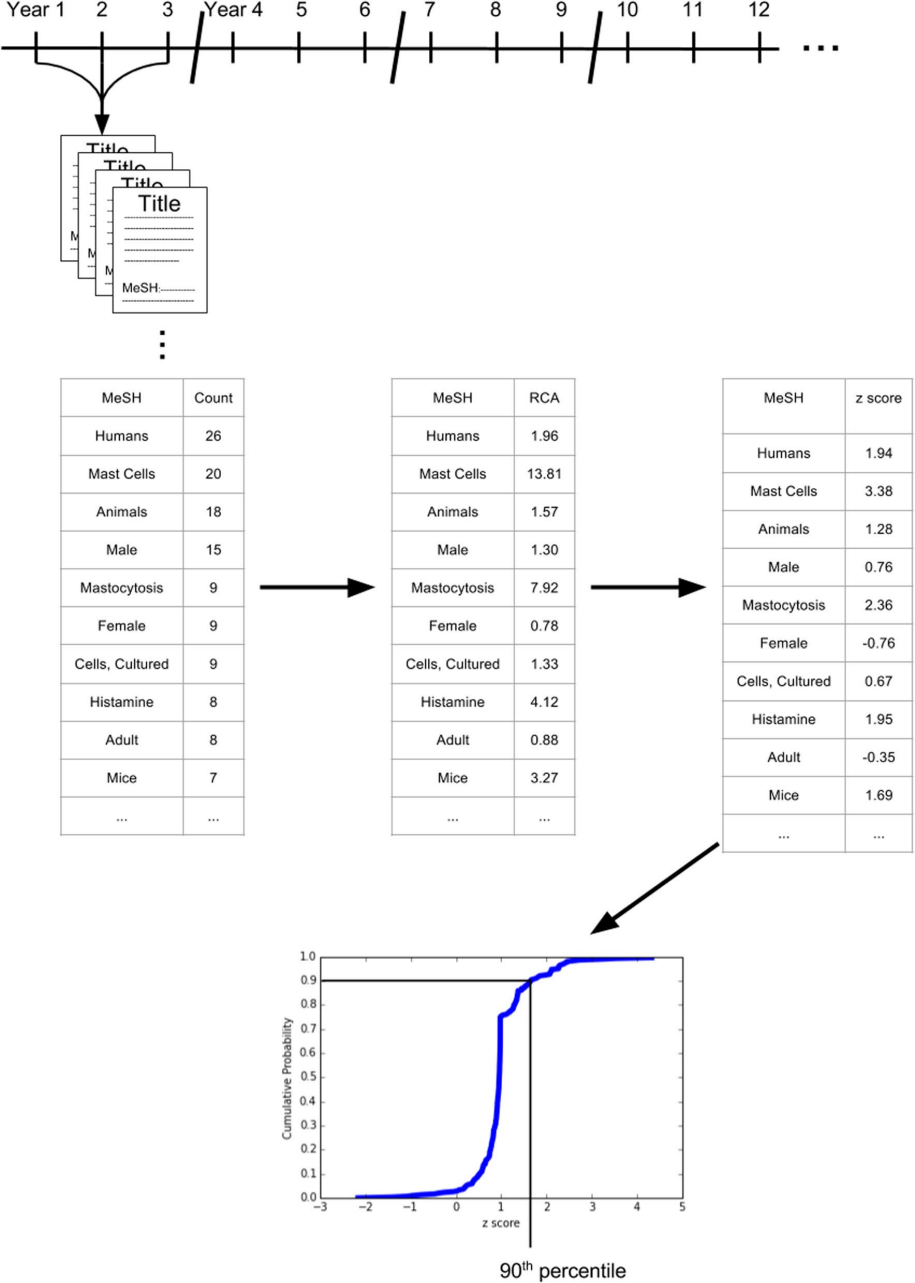
Workflow for calculating the specialization measure. First a researcher’s career is broken into 3-year windows. Publications are extracted for each window. From those publications, the Medical Subject Headings counts extracted, then the revealed comparative advantage calculated for each. Finally, each RCA is transformed into a *z*-score, and the 90th of the researcher’s *z*-score is that researcher’s extent of specialization in that window ($\text{spec}_{i,w}$)

Our method for estimating the extent to which a researcher is specialized is based on Medical Subject Headings (MeSH) as outlined in Fig. 2. MeSH terms are a set of descriptors that make up a controlled vocabulary managed by the US National Institutes of Health (NIH). Each publication in the PubMed database is assigned a set of MeSH terms characterizing its content by an independent indexer at the National Library of Medicine (an institute based at the NIH). MeSH terms have found broad use as topic tags across a wide variety of applications, including the identification of emerging research avenues [61]; the mapping of the medical research landscape [62]; the modeling of medical innovation dynamics [63], measuring research subject boundaries and research similarity [64]; and the construction of disease-symptom networks [65]. We note that the use of MeSH terms for identifying specific relationships between or trends within given research topic is not perfect. However, we suggest that as long as the assignments of similar works are themselves similar, then the intuition behind our specialization measure, described below, will hold.

For each MeSH term assigned to a researcher’s papers in a given time window, we calculate the researcher’s *revealed comparative advantage* (RCA) in that MeSH term:

1 $$\begin{aligned} \text {RCA}_{{i,m,w}} = \frac{n_{i,m,w}}{p_{i,w}} \Big/ \left( \frac{N_{m,w}}{P_{w}} \right) \end{aligned}$$

where $n_{i,m,w}$ is the number of researcher *i*’s papers from time window *w* on which MeSH term *m* appears, and $p_{i,w}$ is the number of papers published by that researcher in the same time window. The variable $N_{m,w}$ is the number of papers in that time window with MeSH term *m* in the PubMed database, and $P_{w}$ is the total number of papers published in that time window. In words, RCA captures the fraction of a researcher’s output that is associated with a particular MeSH heading, relative to the same fraction averaged across all researchers in the cohort.

We then apply a *z*-score transform to the quantity $\text {RCA}_{{i,m,w}}$:

2 $$\begin{aligned} z_{i,m,w} = \frac{\text {RCA}_{{i,m,w}} - 1}{\sigma _{\text {RCA}_{{i,m,w}}}}, \end{aligned}$$

in which the standard deviation of $\text {RCA}_{{i,m,w}}$ is calculated assuming counting statistics (see Appendix for derivation):

3 $$\begin{aligned} \sigma _{\text {RCA}} = \text {RCA} \left( \frac{1}{n} + \frac{1}{p} + \frac{1}{P} + \frac{1}{N}\right) ^{1/2}. \end{aligned}$$

For example, a *z*-score of 1.2 for a specific MeSH term indicates that the MeSH term is assigned to the researcher’s publications 1.2 standard deviations more than would be expected from the global average (within the same time window). Hence, the *z*-score measures how focused the researcher is on specific topics or concepts at various points in their career. For each window of a researcher’s career, we have a *z*-score for each MeSH term assigned to their publications in that window. Treating these *z*-scores as a distribution over MeSH terms for a given individual in a particular time window, our measure of specialization ($\text{spec}_{i,w}$) is the 90th percentile of this distribution. The higher a researcher’s value of $\text{spec}_{i,w}$, the more intensely focused that researcher is upon the topics they are working on the most, relative to other researchers. A lower value indicates that the researcher is more diffused in the topics they are working on the most. In short, the measure concerns only the topics the researcher is working on the most, relative to the effort expended on these topics by other researchers. Researchers can obtain a high score by publishing on several rare topics or a much smaller number of more mainstream topics—specialization is effectively measured relative to the average expertise within the biomedical field.

For example, a researcher whose publications are almost entirely at the intersection of Alzheimer’s disease, electrophysiology, and drug discovery would be considered very specialized (even though they produce highly interdisciplinary work), because the same few MeSH terms appear in many of publications they produce, despite these fields being relatively large. In contrast, a researcher working in the above fields in roughly equal proportions but without much MeSH-term overlap on individual publications would be considered more generalized, even if these publications were relatively monodisciplinary. In this way, a specialist can produce exclusively interdisciplinary work, and a generalist can produce exclusively monodisciplinary work, which distinguishes our measure from those in the extant literature. In reality, while we consider specialization and disciplinarity to be distinct concepts in this work, we expect that there is a strong inverse relationship between the interdisciplinarity of particular publications and the specialization of the authors.

With respect to dynamics, because we calculate specialization in 3-year blocks, the mid- to long-term evolution of the MeSH dictionary have minimal impact on our measure. Furthermore, any effects of short-term popularity dynamics of particular MeSH headings are mitigated by considering the RCA rather than considering only the usage of terms by each individual. For example, the measure can differentiate between a particular researcher starting to study a specific topic more frequently and an entire field doing the same.

**Fig. 3:**
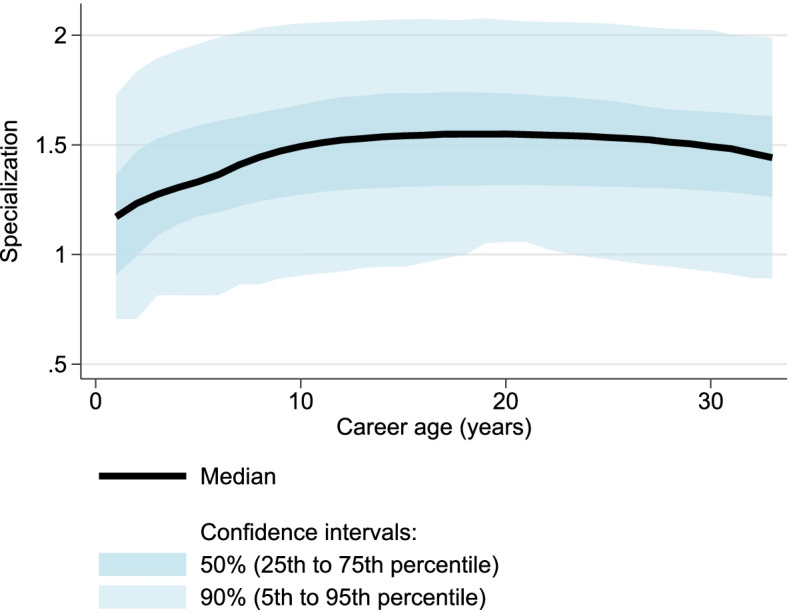
Specialization as a function of career age. Here, the distribution of raw researcher-window specialization values ($\text{spec}_{i,w}$) is plotted as a function of career age. There is a slight increase early in the career, followed by an extended period of minimal variation in the heart of the career. The slight drop after career age 30 may be attributable to the fact that not all careers in our dataset extend beyond that age. Based on 29,197 unique biomedical researchers for a total of 213,019 researcher-time window observations

Figure 3 illustrates the distribution of the specialization measure as a function of career age. It shows a slight increase early in the career, followed by an extended period of minimal variation.

### Regression model

Next, we estimate the extent to which specialization affects a researcher’s scientific impact using an econometric regression model. As researchers are active over many time windows in the dataset (up to ten time windows covering 30 years), we are able to use variation in each researcher’s specialization across time windows as the source for econometric identification. The specific model we estimate is as follows:

4 $$\begin{aligned} I_{i,w}&=\beta _{1} \text{spec}_{i,w} + \beta _{2}(\text{spec}_{i,w} \times p_{i,w}) + \beta _{3} (\text{spec}_{i,w} \times \text{age}_{i,w}) \nonumber \\&\quad + \varvec{\gamma }\mathbf {x}_{\mathbf {i,w}} + \delta _{w} + c_{i} + \epsilon _{i,w} . \end{aligned}$$

where $I_{i,w}$ is the logarithm of the average number of citations accumulated by researcher *i*’s papers published in window *w* up to 5 years after publication.^1^ The variable $\text{spec}_{i,w}$ is the main variable of interest described above (standardized for the regression tables), and career age ($\text{age}_{i,w}$, number of years since first publication), publishing rate ($p_{i,w}$, number of papers published in *w*), and cosine similarity $\text{cosine}_{i,w}$ are secondary variables of interest. The term $\mathbf {x}_{\mathbf {i,w}}$ contains $\text{age}_{i,w}$, $p_{i,w}$, $\text{cosine}_{i,w}$, and additional variables controlling for the number of unique co-authors within a window and the number of unique MeSH terms extracted from the researcher’s papers in each window. The variables $\delta _{w}$ and $c_{i}$ represent time and researcher fixed-effects, while $\epsilon _{i,w}$ is an error term.

The interpretation of the specialization parameter is as follows: a one-standard deviation increase in the specialization measure of the researcher in a given time window is associated with a $\beta _{1}*100$% increase in the number of citations that they received for the papers published in the time window. This time window-based approach also means that researchers who transition between fields appear less specialized only briefly—such transitions do not lead to persistently lower level of observed specialization. Furthermore, because analyses are conducted within-researcher, we do not apply any explicit subject-level citation normalizations.

Cosine similarity, $\text{cosine}_{i,w}$, captures the degree to which a researcher’s topical output has changed since the last window. This variable is calculated as follows:

5 $$\begin{aligned} \text{cosine}_{i,w} = \frac{\sum \nolimits _{j=1}^{n}{M_{i,w}[j] \, M_{i,w-1}[j]} }{ \sqrt{\sum \nolimits _{j=1}^{n}{(M_{i,w}[j])^{2}}} \sqrt{\sum \nolimits _{j=1}^{n}{(M_{i,w-1}[j])^{2}}} } \quad , \end{aligned}$$

where $M_{i,w}$ is a vector wherein the $j^{th}$ entry captures the number of articles that researcher *i* publishes in window *w* that are tagged with the *j*th MeSH term (from the universe of *n* ordered MeSH terms). This value is maximized (equal to unity) when the vectors $M_{i,w}$ and $M_{i,w-1}$ are identical and minimized (equal to 0) when the sets of MeSH represented by $M_{i,w}$ and $M_{i,w-1}$ are disjoint. This variable is set to 0 for the first window during which a researcher is active.

## Figures and Tables

**Fig. 4:**
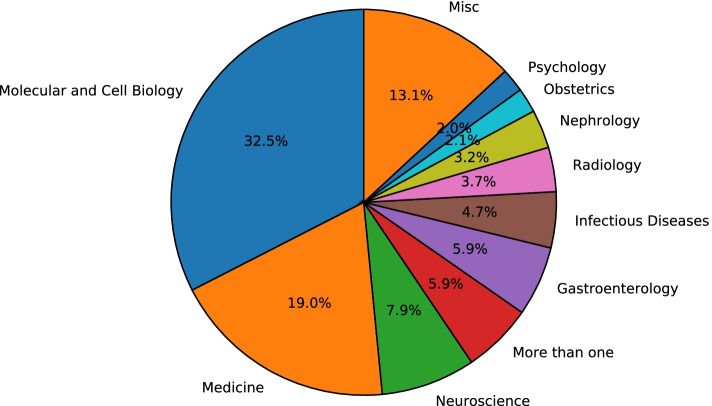
Breakdown of researcher disciplines for our sample of 29,208 researchers with at least 100 publications

**Fig. 5:**
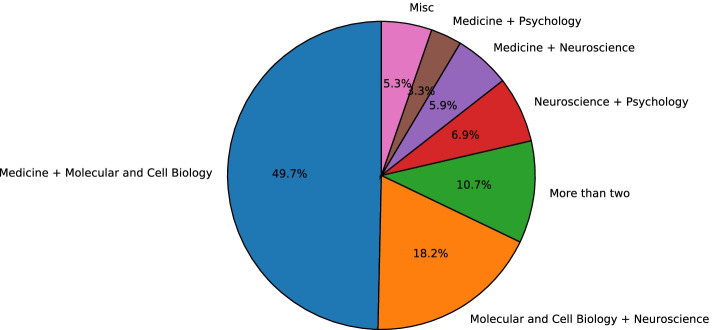
Breakdown of disciplines for researchers assigned to more than one discipline for the associated subsample of 1716 researchers with at least 100 publications

**Fig. 6:**
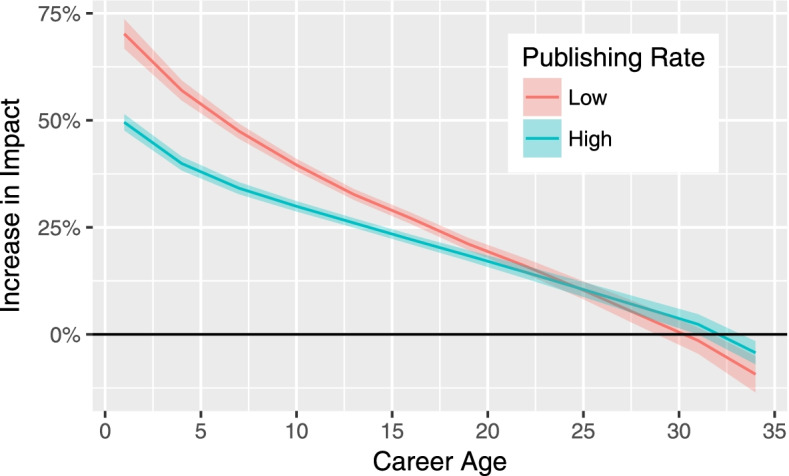
Marginal effects for biomedical researchers with 75 to 99 career publications. Low publishing rate is estimated at the 12.5th percentile (middle of the first quartile) of papers per year. High publishing rate is estimated at the 87.5th percentile (middle of the fourth quartile) of papers per year. The shaded envelope of each line is the $99.9\%$ confidence interval. Based on 22,589 unique biomedical researchers with between 75 and 99 career publications career publications, for a total of 145,143 researcher-time window observations

**Fig. 7:**
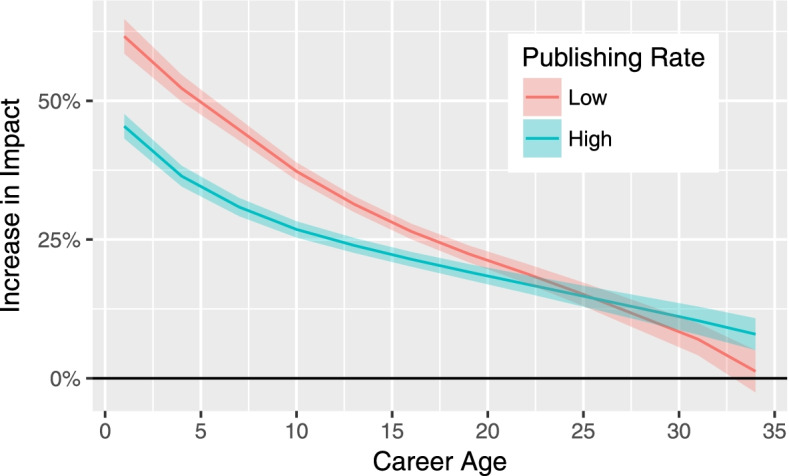
Marginal effects for researchers in molecular and cell biology with more than 100 publications. Low publishing rate is estimated at the 12.5th percentile (middle of the first quartile) of papers per year. High publishing rate is estimated at the 87.5th percentile (middle of the fourth quartile) of papers per year. The shaded envelope of each line is the $99.9\%$ confidence interval. Based on 10,889 unique researchers with a total of 81,398 researcher-time window observations

**Fig. 8:**
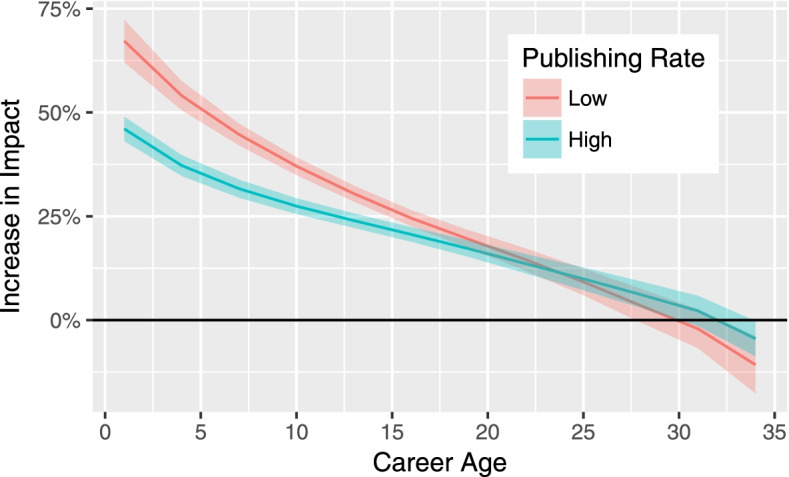
Marginal effects for researchers in molecular and cell biology with 75 to 99 career publications. Low publishing rate is estimated at the 12.5th percentile (middle of the first quartile) of papers per year. High publishing rate is estimated at the 87.5th percentile (middle of the fourth quartile) of papers per year. The shaded envelope of each line is the $99.9\%$ confidence interval. Based on 8135 unique researchers with a total of 53,890 researcher-time window observations

**Fig. 9:**
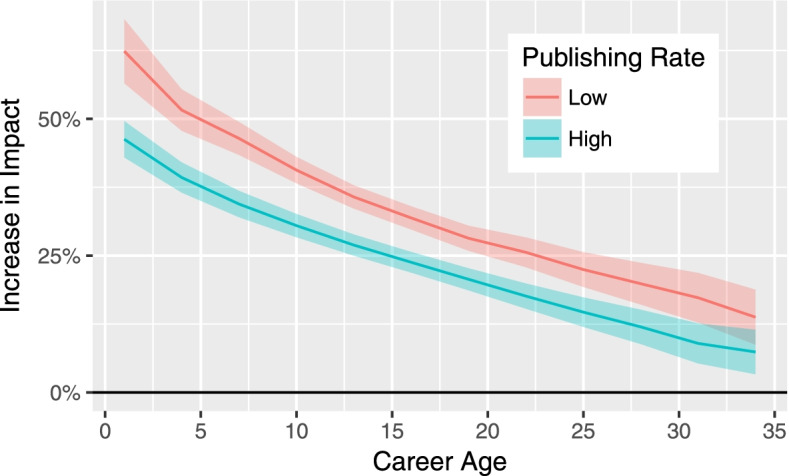
Marginal effects for researchers in medicine with more than 100 publications. Low publishing rate is estimated at the 12.5th percentile (middle of the first quartile) of papers per year. High publishing rate is estimated at the 87.5th percentile (middle of the fourth quartile) of papers per year. The shaded envelope of each line is the $99.9\%$ confidence interval. Based on 6722 unique researchers with a total of 48,433 researcher-time window observations

**Fig. 10:**
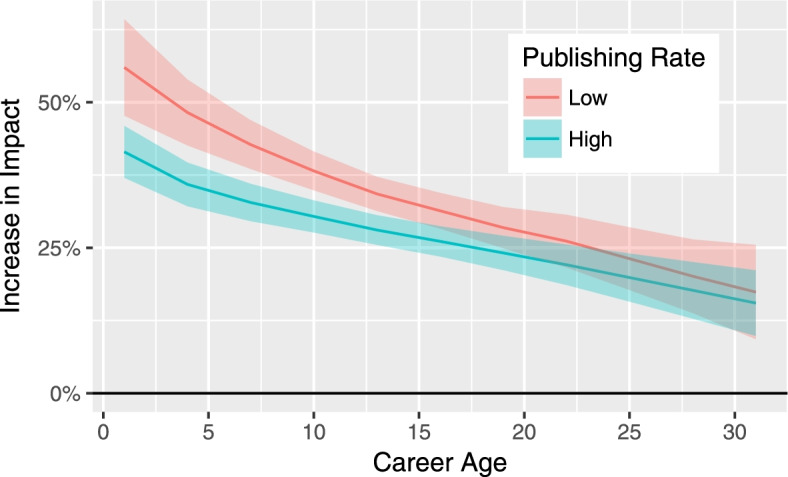
Marginal effects for researchers in medicine with 75 to 99 career publications. Low publishing rate is estimated at the 12.5th percentile (middle of the first quartile) of papers per year. High publishing rate is estimated at the 87.5th percentile (middle of the fourth quartile) of papers per year. The shaded envelope of each line is the $99.9\%$ confidence interval. Based on 4825 unique researchers with a total of 30,440 researcher-time window observations

**Fig. 11:**
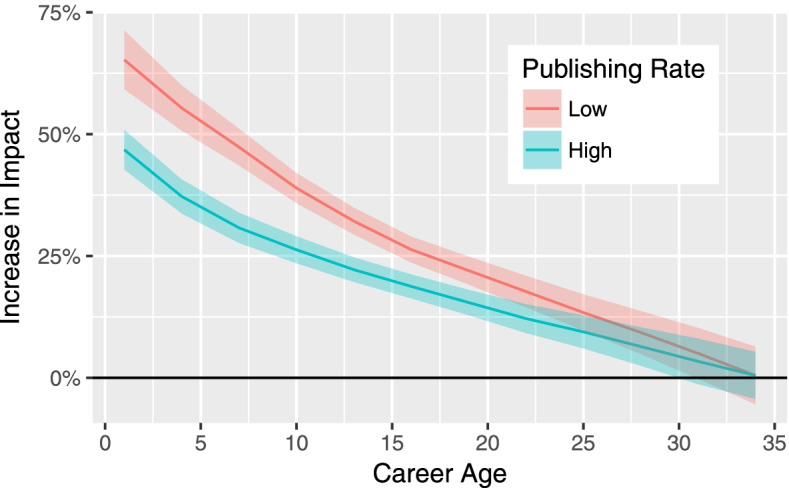
Marginal effects for researchers in neuroscience with 100 or more career publications. Low publishing rate is estimated at the 12.5th percentile (middle of the first quartile) of papers per year. High publishing rate is estimated at the 87.5th percentile (middle of the fourth quartile) of papers per year. The shaded envelope of each line is the $99.9\%$ confidence interval. Based on 2994 unique researchers with a total of 22,006 researcher-time window observations

**Fig. 12:**
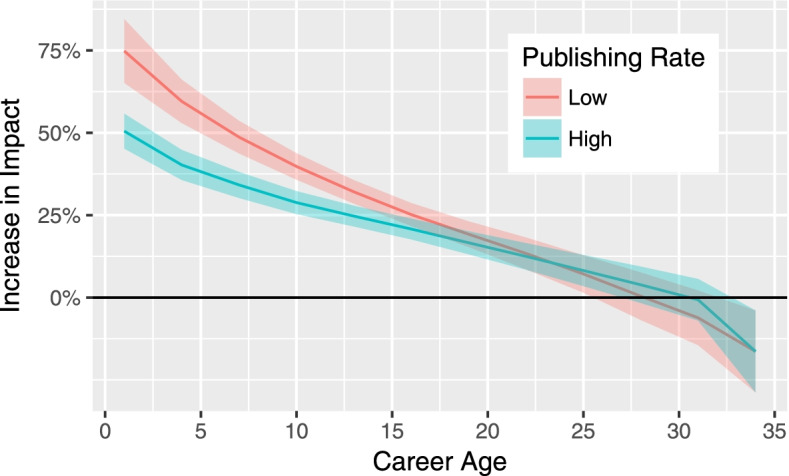
Marginal effects for researchers in neuroscience with between 75 and 99 career publications. Low publishing rate is estimated at the 12.5the percentile (middle of the first quartile) of papers per year. High publishing rate is estimated at the 87.5th percentile (middle of the fourth quartile) of papers per year. The shaded envelope of each line is the $99.9\%$ confidence interval. Based on 2423 unique researchers with a total of 15,992 researcher-time window observations

**Fig. 13:**
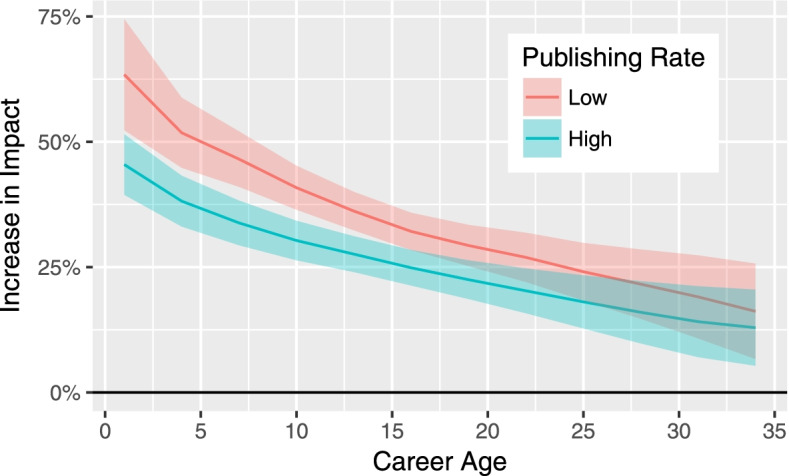
Marginal effects for researchers in gastroenterology with 100 or more career publications. Low publishing rate is estimated at the 12.5th percentile (middle of the first quartile) of papers per year. High publishing rate is estimated at the 87.5th percentile (middle of the fourth quartile) of papers per year. The shaded envelope of each line is the $99.9\%$ confidence interval. Based on 1713 unique researchers with a total of 12,164 researcher-time window observations

**Fig. 14:**
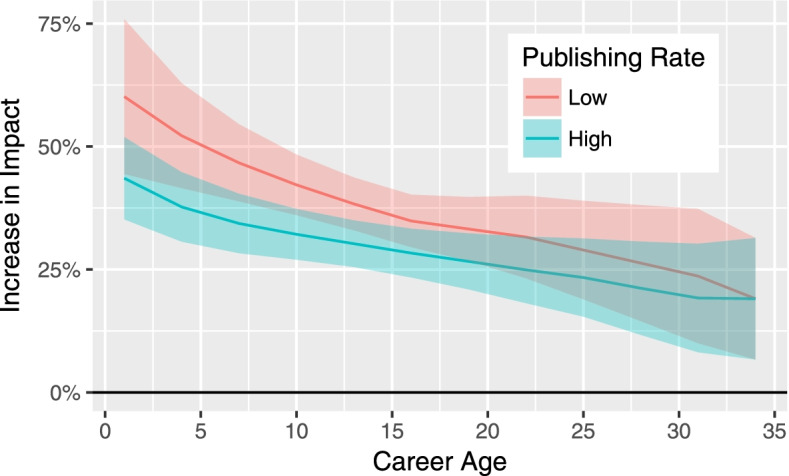
Marginal effects for researchers in gastroenterology with between 75 and 99 career publications. Low publishing rate is estimated at the 12.5th percentile (middle of the first quartile) of papers per year. High publishing rate is estimated at the 87.5th percentile (middle of the fourth quartile) of papers per year. The shaded envelope of each line is the $99.9\%$ confidence interval. Based on 1304 unique researchers with a total of 8017 researcher-time window observations

**Fig. 15:**
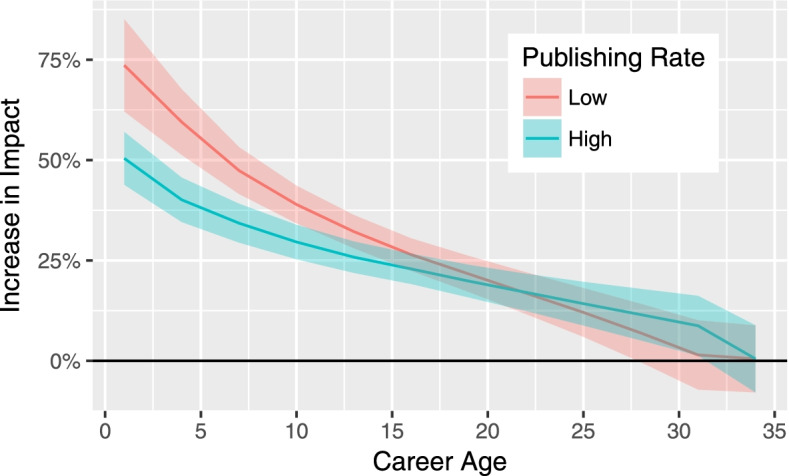
Marginal effects for researchers in infectious diseases with 100 or more career publications. Low publishing rate is estimated at the 12.5th percentile (middle of the first quartile) of papers per year. High publishing rate is estimated at the 87.5th percentile (middle of the fourth quartile) of papers per year. The shaded envelope of each line is the $99.9\%$ confidence interval. Based on 1396 unique researchers with a total of 10,073 researcher-time window observations

**Fig. 16:**
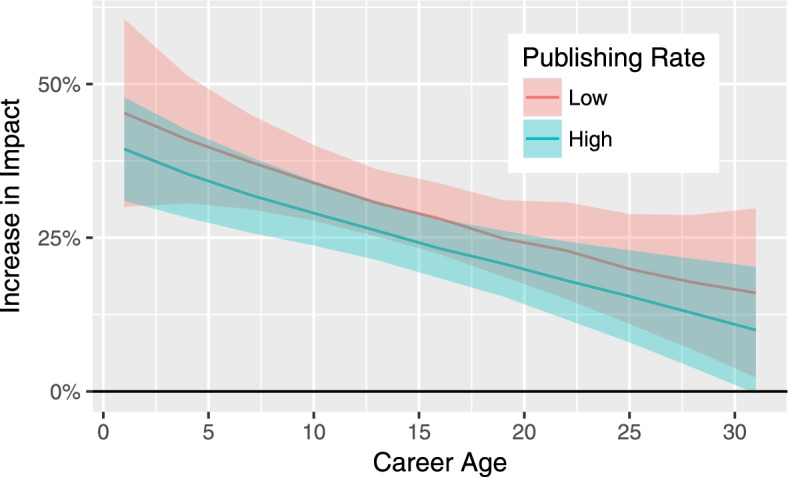
Marginal effects for researchers in infectious diseases with between 75 and 99 career publications. Low publishing rate is estimated at the 12.5th percentile (middle of the first quartile) of papers per year. High publishing rate is estimated at the 87.5th percentile (middle of the fourth quartile) of papers per year. The shaded envelope of each line is the $99.9\%$ confidence interval. Based on 1154 unique researchers with a total of 7460 researcher-time window observations

**Fig. 17:**
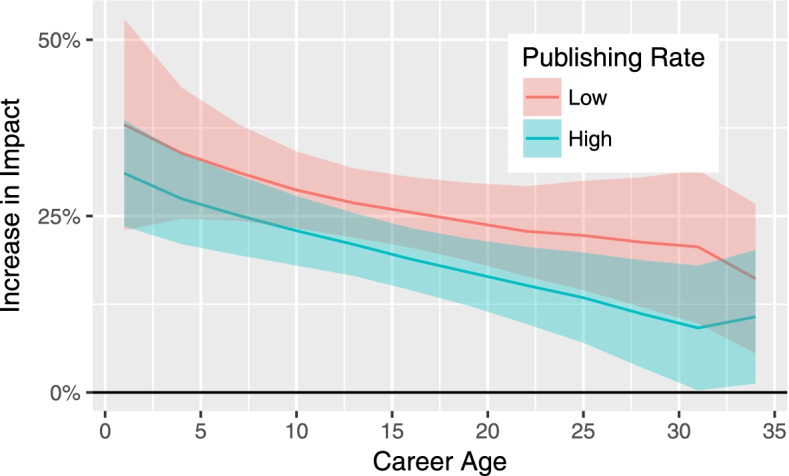
Marginal effects for researchers in radiology with 100 or more career publications. Low publishing rate is estimated at the 12.5th percentile (middle of the first quartile) of papers per year. High publishing rate is estimated at the 87.5th percentile (middle of the fourth quartile) of papers per year. The shaded envelope of each line is the $99.9\%$ confidence interval. Based on 1086 unique researchers with a total of 7757 researcher-time window observations

**Fig. 18:**
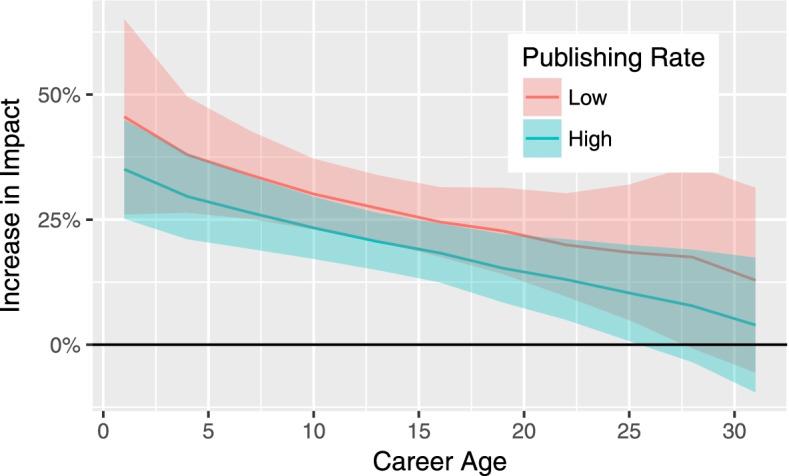
Marginal effects for researchers in radiology with between 75 and 99 career publications. Low publishing rate is estimated at the 12.5th percentile (middle of the first quartile) of papers per year. High publishing rate is estimated at the 87.5th percentile (middle of the fourth quartile) of papers per year. The shaded envelope of each line is the $99.9\%$ confidence interval. Based on 902 unique researchers with a total of 5483 researcher-time window observations

**Fig. 19:**
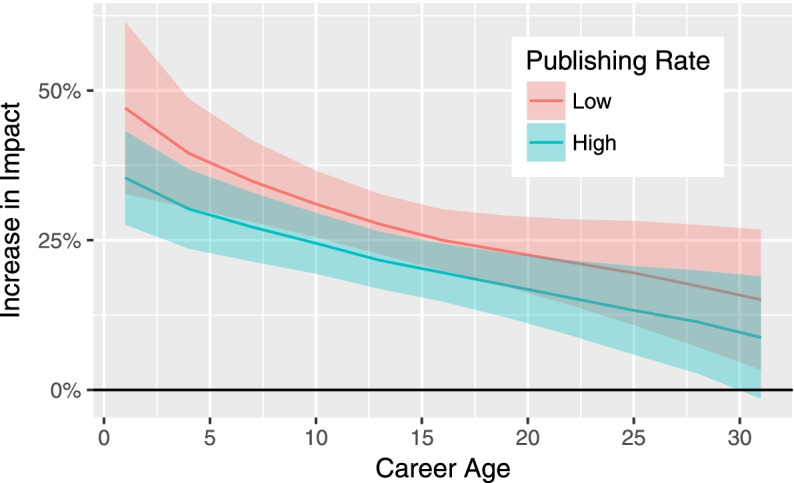
Marginal effects for researchers in nephrology with 100 or more career publications. Low publishing rate is estimated at the 12.5th percentile (middle of the first quartile) of papers per year. High publishing rate is estimated at the 87.5th percentile (middle of the fourth quartile) of papers per year. The shaded envelope of each line is the $99.9\%$ confidence interval. Based on 944 unique researchers with a total of 6738 researcher-time window observations

**Fig. 20:**
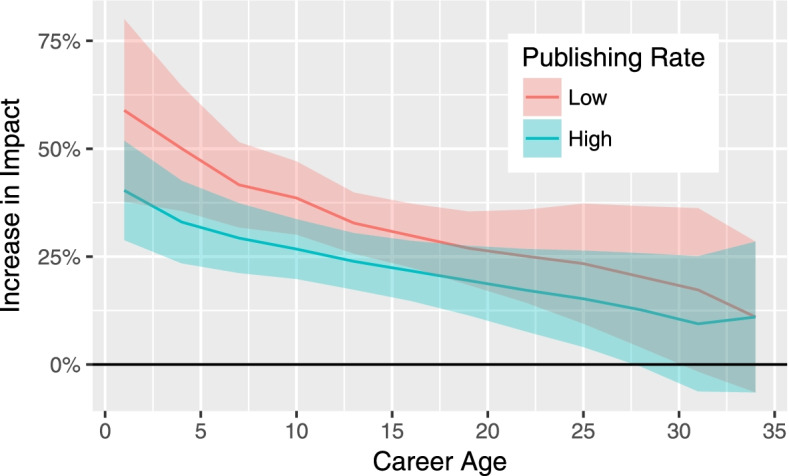
Marginal effects for researchers in nephrology with between 75 and 99 career publications. Low publishing rate is estimated at the 12.5th percentile (middle of the first quartile) of papers per year. High publishing rate is estimated at the 87.5th percentile (middle of the fourth quartile) of papers per year. The shaded envelope of each line is the $99.9\%$ confidence interval. Based on 638 unique researchers with a total of 3962 researcher-time window observations

**Fig. 21:**
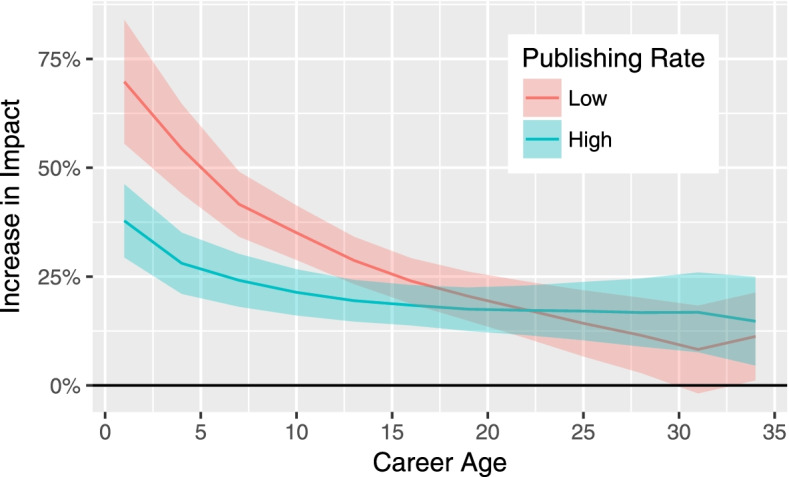
Marginal effects for researchers in psychology with 100 or more career publications. Low publishing rate is estimated at the 12.5th percentile (middle of the first quartile) of papers per year. High publishing rate is estimated at the 87.5th percentile (middle of the fourth quartile) of papers per year. The shaded envelope of each line is the $99.9\%$ confidence interval. Based on 828 unique researchers with a total of 5990 researcher-time window observations

**Fig. 22:**
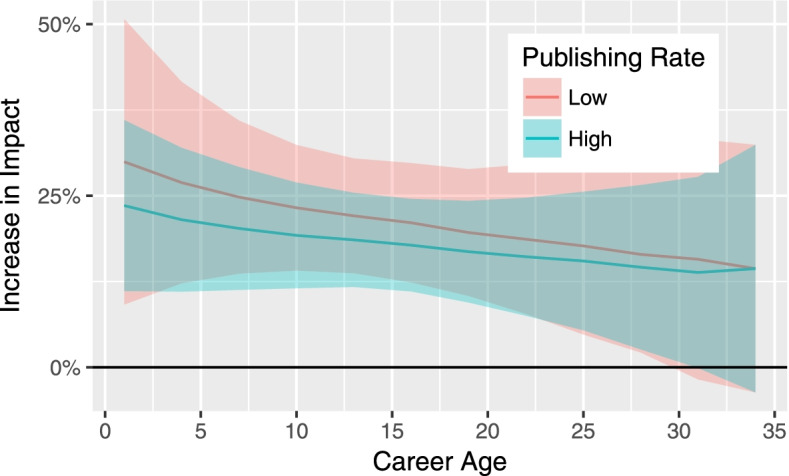
Marginal effects for researchers in psychology with between 75 and 99 career publications. Low publishing rate is estimated at the 12.5th percentile (middle of the first quartile) of papers per year. High publishing rate is estimated at the 87.5th percentile (middle of the fourth quartile) of papers per year. The shaded envelope of each line is the $99.9\%$ confidence interval. Based on 603 unique researchers with a total of 3882 researcher-time window observations

## Data Availability

All data generated or analyzed during this study are included in this published article, its supplementary information files, and publicly available repositories. These data are available in the Zenodo repository (zenodo.org/record/4435704), with DOI identifier 10.5281/zenodo.4435703. Primary data was sourced from Web of Science and PubMed, with additional disambiguation and research discipline assignment conducted and shared by Vetle Torvik (Author-ity) and Jevin West, respectively.

## Appendix

### Discipline breakdown

To better understand the sample of researchers, and also facilitate the discipline by discipline robustness checks, the discipline of each researcher is estimated using the following process. First, the journals a researcher has published in are extracted from his or her publications. In this stage, we also eliminate highly interdisciplinary journals (PNAS, Science, Nature, Annals of the New York Academy of Science, and PLoS ONE). Second, these journal titles are matched to the map equation journal classifications developed by Rosvall and Bergstrom [60]. To assign disciplines, the following algorithm is then followed:

A breakdown of the disciplines assigned can be found in Figs. 4 and 5.

### Derivation of standard deviation of RCA

Starting with the equation for revealed comparative advantage:

6 $$\begin{aligned} \text {RCA}_{{i,m,w}} = \frac{n_{i,m,w}}{p_{i,t}} \Big / \left( \frac{N_{m,w}}{P_{w}} \right) \end{aligned}$$

We note it is an equation of four variables, namely $n_{i,m,w}$, $p_{i,w}$, $N_{m,w}$, and $P_{w}$. Drawing from propagation of uncertainties, we understand that the covariance (*C*) of an arbitrary function *f* can be expressed as follows:

7 $$\begin{aligned} \mathbf {C}_{\mathbf {f}} = \mathbf {J}\mathbf {C}_{\mathbf {x}}\mathbf {J}^{\top } \end{aligned}$$

where *x* in the right hand side covariance matrix ($\mathbf {C}_{\mathbf {x}}$) denotes it is over the independent variables of Eq. 6, and *J* is the Jacobian matrix. Executing this for the $\text {RCA}_{{i,m,w}}$ and assuming no correlation between the independent variables, we get the following relationship:

8 $$\begin{aligned} \sigma ^{2}_{\text {RCA}} = \left| \frac{\partial \text {RCA}}{\partial n} \right| ^{2} \sigma ^{2}_{n} + \left| \frac{\partial \text {RCA}}{\partial p} \right| ^{2} \sigma ^{2}_{p} + \left| \frac{\partial \text {RCA}}{\partial N} \right| ^{2} \sigma ^{2}_{N} + \left| \frac{\partial \text {RCA}}{\partial P} \right| ^{2} \sigma ^{2}_{P}. \end{aligned}$$

It is straightforward to show that this equation simplifies to:

9 $$\begin{aligned} \sigma ^{2}_{\text {RCA}} = \left| \frac{\text {RCA}}{n} \right| ^{2} \sigma ^{2}_{n} + \left| \frac{\text {RCA}}{p} \right| ^{2} \sigma ^{2}_{p} + \left| \frac{\text {RCA}}{N} \right| ^{2} \sigma ^{2}_{N} + \left| \frac{\text {RCA}}{P} \right| ^{2} \sigma ^{2}_{P}. \end{aligned}$$

Making the assumption that the probability that any given MeSH term appears on a paper arises from a binomial distribution, we can use the property $\sigma ^{2}_{x} = x$ to further simplify:

10 $$\begin{aligned} \sigma ^{2}_{\text {RCA}} = \frac{\text {RCA}^{2}}{n^{2}} n + \frac{\text {RCA}^{2}}{p^{2}} p + \frac{\text {RCA}^{2}}{N^{2}} N + \frac{\text {RCA}^{2}}{P^{2}} P. \end{aligned}$$

Canceling common factors and factoring out RCA, we arrive at:

11 $$\begin{aligned} \sigma _{\text {RCA}} = \text {RCA} \left( \frac{1}{n} + \frac{1}{p} + \frac{1}{P} + \frac{1}{N}\right) ^{1/2} \end{aligned}$$

as used in the main manuscript.

### Robustness checks

To check the robustness of our results, we carry out a number of additional regressions. First, we perform the same analysis as found in the main manuscript, but on a different, less published, sample of biomedical researchers. Second, we conduct a non-parametric test to demonstrate the lack of a relationship between our specialization measure and a more general measure of interdisciplinarity. Finally, we carry out the same analysis as in the main manuscript, but on researchers of specified disciplines of the biomedical sciences.

### Lower publishing sample

Here, we carry out the same regression as found in the main manuscript but on a sample of biomedical researchers publishing between 75 and 99 publications over the course of their careers. The results can be found in Table 2 and Fig. 6 and are consistent with the findings in the main body of the paper.

### Specialization and interdisciplinarity

Our measure of specialization captures the diversity of topics on which a scientist is working. As such, if a scientist is working on a small set of topics that are spread across traditionally defined fields, this person would be considered to be specialized *and* interdisciplinary. For this reason, in the main body of the paper, we claim that the opposite of specialization is not interdisciplinarity but rather generalization.

Here, we conduct simple statistical tests to demonstrate the lack of relationship between our specialization measure and a measure of interdisciplinarity. For this purpose, we consider a researcher to be interdisciplinary if they have been assigned multiple disciplines (as defined above) where, importantly, these disciplines are defined independently from MeSH terms. This definition is quite strict, which provides some assurance that these interdisciplinary researchers have indeed published a significant number of papers in multiple disciplines throughout their career. However, we also accept that this could occur when a researcher moves between disciplines rather than working across disciplines. Therefore, to give the best possible chance for a significant difference in specialization to be found between interdisciplinary and non-interdisciplinary researchers, we consider both the minimum and the average levels of specialization for each researcher throughout their career. This precaution will pick up a transition between disciplines as the least specialized period of the researcher’s career, and taken alone, this period would be challenging to distinguish from “true” generalization (as opposed to a transient state).

If generalization and interdisciplinarity were significantly correlated, we would expect the least specialized period in interdisciplinary researchers’ careers to be lower than that of non-interdisciplinary researchers. We may also expect their average specialization to be lower. Furthermore, as average specialization levels do not appear to stabilize until about 10 years into a career, we may wish to consider this latter part of the career separately. As such, we test all four of these scenarios for significant differences between interdisciplinary researchers and non-interdisciplinary researchers: minimum specialization across the whole career, minimum specialization for career age greater than 10 years, average specialization across the whole career, and average specialization for career age greater than 10 years. We use two non-parametric tests for this purpose: the Mann-Whitney *U* test and the two-sample Kolmogorov-Smirnov test. The former calculates the probability that a randomly chosen interdisciplinary researcher is less specialized than a randomly chosen non-interdisciplinary researcher, while the latter directly compares the cumulative specialization distributions of each group and tests the significance of any differences. We conduct these tests for all researchers in the primary cohort (greater than 100 publications) for whom we were able to obtain sufficient information about disciplines (not assigned “NULL”). In total, 29,670 researchers are included in this analysis, of which 1709 (5.8%) are classified as interdisciplinary.

Table 3 displays the results of these tests. The *p*-values for all tests indicate that any differences in specialization levels between interdisciplinary researchers and non-interdisciplinary researchers are not significant. This result is consistent with our assertion that the specialization measure does *not* measure interdisciplinarity (or lack thereof), at least for biomedical researchers with long careers. While not displayed here, the same (qualitative) results are found when the threshold of the distribution over MeSH terms, used to obtain our specialization measure, is set to 80% or 95%.

### Separate disciplines

To again better understand the robustness of our findings, we carry out the same regression analysis as in the main manuscript for each of the eight most common disciplines in our dataset of researchers. Disciplines are assigned according to the procedure outlined in Section 1 above. Note that these regressions include *all* researchers assigned to each specific discipline, and hence, a research assigned to multiple disciplines will appear in more than one regression. For each discipline, we further report results for both the standard sample consisting of researcher publishing 100 or more papers in their career, as well as the set of researchers publishing 75 to 99 papers. These results are presented in Tables 4, 5, 6, 7, 8, 9, 10, 11, 12, 13, 14, 15, 16, 17, 18, and 19 and Figs. 7, 8, 9, 10, 11, 12, 13, 14, 15, 16, 17, 18, 19, 20, 21, and 22.

**Table 2:** Fixed-effects panel regression results. Dependent variable is the log number of citations per paper, and the “specialization” variable is standardized. Standard errors are in parentheses. All control variables described in the main manuscript are included. Based on 22,589 unique biomedical researchers with between 75 and 99 career publications career publications, for a total of 145,143 researcher-time window observations

	(1)	(2)	(3)	(4)
Specialization	0.265***	0.359***	0.410***	0.727***
	(0.004)	(0.006)	(0.008)	(0.014)
Specialization × career age		− 0.007***		− 0.025***
		(0.0003)		(0.001)
Specialization × papers			− 0.055***	− 0.098***
			(0.002)	(0.005)
Career age × papers				0.021***
				(0.001)
Specialization × career age × papers				0.004***
				(0.0003)
Observations	145,143	145,143	145,143	145,143
*R* ^2^	0.174	0.178	0.178	0.186
Adjusted *R*^2^	0.022	0.026	0.026	0.036
Control variables	Yes	Yes	Yes	Yes
Year fixed-effects	Yes	Yes	Yes	Yes
Researcher fixed-effects	Yes	Yes	Yes	Yes

**p *< 0.01; ***p *< 0.001; ****p *< 0.0001

**Table 3:** *p*-values from two non-parametric tests of the differences in specialization between interdisciplinary researchers and non-interdisciplinary researchers. M-W corresponds to the Mann-Whitney *U* test, while K-S corresponds to the two-sample Kolmogorov-Smirnov test. No significant differences are found between the two groups at any conventional *p*-value threshold. Analysis conducted for the sample of 29,208 researchers with at least 100 publications

	M-W test	K-S test
	All years (*p*-values)	
Min. specialization	0.97	0.78
Ave. specialization	0.60	0.47
	> 10 years (*p*-values)	
Min. specialization	0.24	0.50
Ave. specialization	0.17	0.15

**Table 4:** Fixed-effects panel regression results. Dependent variable is the log number of citations per paper, and the “specialization” variable is standardized. Standard errors are in parentheses. All control variables described in the main manuscript are included. Based on 10,889 unique biomedical researchers assigned to the discipline molecular and cell biology with at least 100 publications

	(1)	(2)	(3)	(4)
Specialization	0.251***	0.323***	0.422***	0.708***
	(0.005)	(0.007)	(0.009)	(0.015)
Specialization × career age		− 0.005***		− 0.022***
		(0.0003)		(0.001)
Specialization × papers			− 0.057***	− 0.105***
			(0.002)	(0.004)
Career age × papers				0.015***
				(0.001)
Specialization × career age × papers				0.004***
				(0.0003)
Observations	81,398	81,398	81,398	81,398
*R* ^2^	0.163	0.166	0.169	0.177
Adjusted *R*^2^	0.033	0.037	0.040	0.049
Control variables	Yes	Yes	Yes	Yes
Year fixed-effects	Yes	Yes	Yes	Yes
Researcher fixed-effects	Yes	Yes	Yes	Yes

**p *< 0.01; ***p *< 0.001; ****p *< 0.0001

**Table 5:** Fixed-effects panel regression results. Dependent variable is the log number of citations per paper, and the “specialization” variable is standardized. Standard errors are in parentheses. All control variables described in the main manuscript are included. Based on 8135 unique biomedical researchers assigned to the discipline molecular and cell biology with between 75 and 99 career publications

	(1)	(2)	(3)	(4)
Specialization	0.249***	0.342***	0.395***	0.696***
	(0.006)	(0.009)	(0.012)	(0.021)
Specialization × career age		− 0.007***		− 0.025***
		(0.0004)		(0.001)
Specialization × papers			− 0.057***	− 0.100***
			(0.004)	(0.007)
Career age × papers				0.020***
				(0.001)
Specialization × career age × papers				0.004***
				(0.001)
Observations	53,890	53,890	53,890	53,890
*R* ^2^	0.133	0.138	0.137	0.146
Adjusted *R*^2^	− 0.022	− 0.016	− 0.017	− 0.007
Control variables	Yes	Yes	Yes	Yes
Year fixed-effects	Yes	Yes	Yes	Yes
Researcher fixed-effects	Yes	Yes	Yes	Yes

**p *< 0.01; ***p *< 0.001; ****p *< 0.0001

**Table 6:** Fixed-effects panel regression results. Dependent variable is the log number of citations per paper, and the “specialization” variable is standardized. Standard errors are in parentheses. All control variables described in the main manuscript are included. Based on 6722 unique biomedical researchers assigned to the discipline medicine with at least 100 publications

	(1)	(2)	(3)	(4)
Specialization	0.275***	0.365***	0.478***	0.636***
	(0.007)	(0.010)	(0.013)	(0.024)
Specialization × career age		− 0.006***		− 0.012***
		(0.0005)		(0.001)
Specialization × papers			− 0.064***	− 0.068***
			(0.003)	(0.007)
Career age × papers				0.011***
				(0.001)
Specialization × career age × papers				0.001
				(0.0004)
Observations	48,433	48,433	48,433	48,433
*R* ^2^	0.261	0.264	0.267	0.270
Adjusted *R*^2^	0.141	0.144	0.148	0.151
Control variables	Yes	Yes	Yes	Yes
Year fixed-effects	Yes	Yes	Yes	Yes
Researcher fixed-effects	Yes	Yes	Yes	Yes

**p *< 0.01; ***p *< 0.001; ****p *< 0.0001

**Table 7:** Fixed-effects panel regression results. Dependent variable is the log number of citations per paper, and the “specialization” variable is standardized. Standard errors are in parentheses. All control variables described in the main manuscript are included. Based on 4825 unique biomedical researchers assigned to the discipline medicine with between 75 and 99 career publications

	(1)	(2)	(3)	(4)
Specialization	0.292***	0.343***	0.411***	0.572***
	(0.010)	(0.013)	(0.018)	(0.034)
Specialization × career age		− 0.004***		− 0.012***
		(0.001)		(0.002)
Specialization × papers			− 0.044***	− 0.065***
			(0.006)	(0.011)
Career age × papers				0.010***
				(0.002)
Specialization × career age × papers				0.002
				(0.001)
Observations	30,440	30,440	30,440	30,440
*R* ^2^	0.217	0.218	0.219	0.220
Adjusted *R*^2^	0.068	0.069	0.070	0.072
Control variables	Yes	Yes	Yes	Yes
Year fixed-effects	Yes	Yes	Yes	Yes
Researcher fixed-effects	Yes	Yes	Yes	Yes

**p *< 0.01; ***p *< 0.001; ****p *< 0.0001

**Table 8:** Fixed-effects panel regression results. Dependent variable is the log number of citations per paper, and the “specialization” variable is standardized. Standard errors are in parentheses. All control variables described in the main manuscript are included. Based on 2994 unique biomedical researchers assigned to the discipline neuroscience with at least 100 publications

	(1)	(2)	(3)	(4)
Specialization	0.225***	0.365***	0.479***	0.749***
	(0.009)	(0.013)	(0.017)	(0.029)
Specialization × career age		− 0.009***		− 0.022***
		(0.001)		(0.002)
Specialization × papers			− 0.082***	− 0.111***
			(0.005)	(0.009)
Career age × papers				0.013***
				(0.001)
Specialization × career age × papers				0.003***
				(0.001)
Observations	22,006	22,006	22,006	22,006
*R* ^2^	0.219	0.229	0.231	0.240
Adjusted *R*^2^	0.094	0.106	0.108	0.119
Control variables	Yes	Yes	Yes	Yes
Year fixed-effects	Yes	Yes	Yes	Yes
Researcher fixed-effects	Yes	Yes	Yes	Yes

**p *< 0.01; ***p *< 0.001; ****p *< 0.0001

**Table 9:** Fixed-effects panel regression results. Dependent variable is the log number of citations per paper, and the “specialization” variable is standardized. Standard errors are in parentheses. All control variables described in the main manuscript are included. Based on 2423 unique biomedical researchers assigned to the discipline neuroscience with between 75 and 99 career publications

	(1)	(2)	(3)	(4)
Specialization	0.245***	0.373***	0.415***	0.777***
	(0.012)	(0.016)	(0.022)	(0.040)
Specialization × career age		− 0.009***		− 0.029***
		(0.001)		(0.003)
Specialization × papers			− 0.065***	− 0.116***
			(0.007)	(0.014)
Career age × papers				0.022***
				(0.002)
Specialization × career age × papers				0.005***
				(0.001)
Observations	15,992	15,992	15,992	15,992
*R* ^2^	0.175	0.183	0.180	0.193
Adjusted *R*^2^	0.026	0.034	0.031	0.046
Control variables	Yes	Yes	Yes	Yes
Year fixed-effects	Yes	Yes	Yes	Yes
Researcher fixed-effects	Yes	Yes	Yes	Yes

**p *< 0.01; ***p *< 0.001; ****p *< 0.0001

**Table 10:** Fixed-effects panel regression results. Dependent variable is the log number of citations per paper, and the “specialization” variable is standardized. Standard errors are in parentheses. All control variables described in the main manuscript are included. Based on 1713 unique biomedical researchers assigned to the discipline gastroenterology with at least 100 publications

	(1)	(2)	(3)	(4)
Specialization	0.294***	0.396***	0.492***	0.647***
	(0.013)	(0.018)	(0.025)	(0.045)
Specialization × career age		− 0.007***		− 0.013***
		(0.001)		(0.003)
Specialization × papers			− 0.065***	− 0.076***
			(0.007)	(0.014)
Career age × papers				0.007***
				(0.002)
Specialization × career age × papers				0.002
				(0.001)
Observations	12,164	12,164	12,164	12,164
*R* ^2^	0.294	0.298	0.300	0.303
Adjusted *R*^2^	0.176	0.181	0.183	0.186
Control variables	Yes	Yes	Yes	Yes
Year fixed-effects	Yes	Yes	Yes	Yes
Researcher fixed-effects	Yes	Yes	Yes	Yes

**p *< 0.01; ***p *< 0.001; ****p *< 0.0001

**Table 11:** Fixed-effects panel regression results. Dependent variable is the log number of citations per paper, and the “specialization” variable is standardized. Standard errors are in parentheses. All control variables described in the main manuscript are included. Based on 1304 unique biomedical researchers assigned to the discipline gastroenterology with between 75 and 99 career publications

	(1)	(2)	(3)	(4)
Specialization	0.324***	0.380***	0.471***	0.612***
	(0.018)	(0.026)	(0.034)	(0.065)
Specialization × career age		− 0.004*		− 0.011
		(0.001)		(0.004)
Specialization × papers			− 0.056***	− 0.074**
			(0.011)	(0.022)
Career age × papers				0.007
				(0.003)
Specialization × career age × papers				0.001
				(0.001)
Observations	8,017	8,017	8,017	8,017
*R* ^2^	0.260	0.261	0.263	0.264
Adjusted *R*^2^	0.112	0.113	0.116	0.117
Control variables	Yes	Yes	Yes	Yes
Year fixed-effects	Yes	Yes	Yes	Yes
Researcher fixed-effects	Yes	Yes	Yes	Yes

**p *< 0.01; ***p *< 0.001; ****p *< 0.0001

**Table 12:** Fixed-effects panel regression results. Dependent variable is the log number of citations per paper, and the “specialization” variable is standardized. Standard errors are in parentheses. All control variables described in the main manuscript are included. Based on 1396 unique biomedical researchers assigned to the discipline infectious diseases with at least 100 publications

	(1)	(2)	(3)	(4)
Specialization	0.264***	0.395***	0.436***	0.763***
	(0.014)	(0.020)	(0.027)	(0.047)
Specialization × career age		− 0.009***		− 0.027***
		(0.001)		(0.003)
Specialization × papers			− 0.057***	− 0.105***
			(0.007)	(0.014)
Career age × papers				0.013***
				(0.002)
Specialization × career age × papers				0.005***
				(0.001)
Observations	10,073	10,073	10,073	10,073
*R* ^2^	0.262	0.269	0.267	0.276
Adjusted *R*^2^	0.140	0.149	0.146	0.156
Control variables	Yes	Yes	Yes	Yes
Year fixed-effects	Yes	Yes	Yes	Yes
Researcher fixed-effects	Yes	Yes	Yes	Yes

**p *< 0.01; ***p *< 0.001; ****p *< 0.0001

**Table 13:** Fixed-effects panel regression results. Dependent variable is the log number of citations per paper, and the “specialization” variable is standardized. Standard errors are in parentheses. All control variables described in the main manuscript are included. Based on 1154 unique biomedical researchers assigned to the discipline infectious diseases with between 75 and 99 career publications

	(1)	(2)	(3)	(4)
Specialization	0.263***	0.326***	0.357***	0.461***
	(0.018)	(0.026)	(0.034)	(0.063)
Specialization × career age		− 0.004**		− 0.008
		(0.001)		(0.004)
Specialization × papers			− 0.036*	− 0.026
			(0.011)	(0.021)
Career age × papers				0.009*
				(0.003)
Specialization × career age × papers				− 0.0004
				(0.001)
Observations	7460	7460	7460	7460
*R* ^2^	0.225	0.226	0.226	0.228
Adjusted *R*^2^	0.078	0.079	0.079	0.082
Control variables	Yes	Yes	Yes	Yes
Year fixed-effects	Yes	Yes	Yes	Yes
Researcher fixed-effects	Yes	Yes	Yes	Yes

**p *< 0.01; ***p *< 0.001; ****p *< 0.0001

**Table 14:** Fixed-effects panel regression results. Dependent variable is the log number of citations per paper, and the “specialization” variable is standardized. Standard errors are in parentheses. All control variables described in the main manuscript are included. Based on 1086 unique biomedical researchers assigned to the discipline radiology with at least 100 publications

	(1)	(2)	(3)	(4)
Specialization	0.216***	0.311***	0.379***	0.382***
	(0.016)	(0.023)	(0.033)	(0.061)
Specialization × career age		− 0.006***		− 0.002
		(0.001)		(0.004)
Specialization × papers			− 0.053***	− 0.027
			(0.009)	(0.019)
Career age × papers				− 0.0004
				(0.002)
Specialization × career age × papers				− 0.001
				(0.001)
Observations	7757	7757	7757	7757
*R* ^2^	0.269	0.273	0.273	0.275
Adjusted *R*^2^	0.146	0.150	0.150	0.152
Control variables	Yes	Yes	Yes	Yes
Year fixed-effects	Yes	Yes	Yes	Yes
Researcher fixed-effects	Yes	Yes	Yes	Yes

**p *< 0.01; ***p *< 0.001; ****p *< 0.0001

**Table 15:** Fixed-effects panel regression results. Dependent variable is the log number of citations per paper, and the “specialization” variable is standardized. Standard errors are in parentheses. All control variables described in the main manuscript are included. Based on 902 unique biomedical researchers assigned to the discipline radiology with between 75 and 99 career publications

	(1)	(2)	(3)	(4)
Specialization	0.223***	0.288***	0.344***	0.465***
	(0.021)	(0.030)	(0.042)	(0.080)
Specialization × career age		− 0.005*		− 0.009
		(0.002)		(0.005)
Specialization × papers			− 0.045**	− 0.044
			(0.013)	(0.027)
Career age × papers				0.007
				(0.004)
Specialization × career age × papers				− 0.0001
				(0.002)
Observations	5483	5483	5483	5483
*R* ^2^	0.212	0.214	0.214	0.217
Adjusted *R*^2^	0.050	0.052	0.052	0.055
Control variables	Yes	Yes	Yes	Yes
Year fixed-effects	Yes	Yes	Yes	Yes
Researcher fixed-effects	Yes	Yes	Yes	Yes

**p *< 0.01; ***p *< 0.001; ****p *< 0.0001

**Table 16:** Fixed-effects panel regression results. Dependent variable is the log number of citations per paper, and the “specialization” variable is standardized. Standard errors are in parentheses. All control variables described in the main manuscript are included. Based on 944 unique biomedical researchers assigned to the discipline nephrology with at least 100 publications

	(1)	(2)	(3)	(4)
Specialization	0.236***	0.292***	0.378***	0.479***
	(0.018)	(0.023)	(0.034)	(0.059)
Specialization × career age		− 0.004**		− 0.008
		(0.001)		(0.004)
Specialization × papers			− 0.046***	− 0.047*
			(0.009)	(0.017)
Career age × papers				0.007*
				(0.002)
Specialization × career cge × papers				0.0004
				(0.001)
Observations	6738	6738	6738	6738
*R* ^2^	0.266	0.268	0.270	0.271
Adjusted *R*^2^	0.142	0.144	0.146	0.147
Control variables	Yes	Yes	Yes	Yes
Year fixed-effects	Yes	Yes	Yes	Yes
Researcher fixed-effects	Yes	Yes	Yes	Yes

**p *< 0.01; ***p *< 0.001; ****p *< 0.0001

**Table 17:** Fixed-effects panel regression results. Dependent variable is the log number of citations per paper, and the “specialization” variable is standardized. Standard errors are in parentheses. All control variables described in the main manuscript are included. Based on 638 unique biomedical researchers assigned to the discipline nephrology with between 75 and 99 career publications

	(1)	(2)	(3)	(4)
Specialization	0.266***	0.329***	0.446***	0.600***
	(0.024)	(0.035)	(0.046)	(0.087)
Specialization × career age		− 0.005		− 0.012
		(0.002)		(0.006)
Specialization × papers			− 0.068***	− 0.082*
			(0.015)	(0.028)
Career age × papers				0.009
				(0.004)
Specialization × career age × papers				0.001
				(0.002)
Observations	3962	3962	3962	3962
*R* ^2^	0.246	0.248	0.251	0.253
Adjusted *R*^2^	0.093	0.095	0.099	0.101
Control variables	Yes	Yes	Yes	Yes
Year fixed-effects	Yes	Yes	Yes	Yes
Researcher fixed-effects	Yes	Yes	Yes	Yes

**p *< 0.01; ***p *< 0.001; ****p *< 0.0001

**Table 18:** Fixed-effects panel regression results. Dependent variable is the log number of citations per paper, and the “specialization” variable is standardized. Standard errors are in parentheses. All control variables described in the main manuscript are included. Based on 828 unique biomedical researchers assigned to the discipline psychology with at least 100 publications

	(1)	(2)	(3)	(4)
Specialization	0.212***	0.328***	0.428***	0.722***
	(0.018)	(0.024)	(0.031)	(0.058)
Specialization × career age		− 0.008***		− 0.025***
		(0.001)		(0.004)
Specialization × papers			− 0.068***	− 0.139***
			(0.008)	(0.016)
Career age × papers				0.005
				(0.002)
Specialization × career age × papers				0.006***
				(0.001)
Observations	5990	5990	5990	5990
*R* ^2^	0.338	0.344	0.347	0.354
Adjusted *R*^2^	0.227	0.234	0.237	0.245
Control variables	Yes	Yes	Yes	Yes
Year fixed-effects	Yes	Yes	Yes	Yes
Researcher fixed-effects	Yes	Yes	Yes	Yes

**p *< 0.01; ***p *< 0.001; ****p *< 0.0001

**Table 19:** Fixed-effects panel regression results. Dependent variable is the log number of citations per paper, and the “specialization” variable is standardized. Standard errors are in parentheses. All control variables described in the main manuscript are included. Based on 603 unique biomedical researchers assigned to the discipline psychology with between 75 and 99 career publications

	(1)	(2)	(3)	(4)
Specialization	0.186***	0.233***	0.259***	0.303**
	(0.026)	(0.036)	(0.046)	(0.085)
Specialization × career age		− 0.003		− 0.004
		(0.002)		(0.006)
Specialization × papers			− 0.026	− 0.028
			(0.014)	(0.026)
Career age × papers				0.001
				(0.004)
Specialization × career age × papers				0.0005
				(0.002)
Observations	3882	3882	3882	3882
*R* ^2^	0.240	0.240	0.241	0.241
Adjusted *R*^2^	0.091	0.092	0.092	0.092
Control variables	Yes	Yes	Yes	Yes
Year fixed-effects	Yes	Yes	Yes	Yes
Researcher fixed-effects	Yes	Yes	Yes	Yes

**p *< 0.01; ***p *< 0.001; ****p *< 0.0001

Even though these disciplines span a wide range of subject matters, norms, sample sizes, and career/laboratory structures (e.g., hospital-based clinical research vs. university experimental labs), the results across all are qualitatively consistent with those presented in the main manuscript.

## Abbreviations

RCA: Revealed comparative advantage
MeSH: Medical Subject Headings
NIH: National Institutes of Health (United States)

## Footnotes

1 The citation count for each publication is calculated by summing over its first 5 years (and not the 5 years from the center of the window).
